# An L-Shaped Sparse Array with Reduced Sensitivity to Parametric Mutual Coupling for 2D DOA Estimation

**DOI:** 10.3390/s26196015

**Published:** 2026-09-23

**Authors:** Xiaoming Wu, Wencan Cheng, Fengtong Mei, Yazhou Yang, Yifeng Wu

**Affiliations:** 1 Qianyuan Laboratory, Hangzhou 310013, China; xd.wxm@163.com; 2 Hangzhou Research Institute of Xidian University, Hangzhou 311231, China; chengwencan825@163.com (W.C.); meifengtong@xidian.edu.cn (F.M.); yazhouxgw@163.com (Y.Y.); 3 School of Electronics and Communication Engineering, Sun Yat-sen University, Shenzhen 518107, China

**Keywords:** L-shaped sparse array, mutual coupling, two-dimensional DOA estimation, MUSIC, degrees of freedom

## Abstract

Mutual coupling among array receiving antennas in passive detection systems can introduce amplitude and phase inconsistencies between antenna elements. The resulting amplitude and phase errors across receiving channels not only affect the spectral peak values in subsequent MUSIC-based direction finding, but also cause shifts in the locations of the spectral peaks, thereby increasing detection errors. To reduce geometry-level sensitivity to this effect, this paper presents an analytically constructed L-shaped sparse array for two-dimensional direction-of-arrival (2D DOA) estimation. The array comprises two orthogonal three-subarray branches that share the sensor at the origin. The electrical angle of one branch is first estimated by a one-dimensional (1D) spatial spectrum; the corresponding electrical angle of the other branch is then obtained from a source-conditioned 1D spectrum, yielding paired 2D directional parameters. With 23 physical sensors, the proposed geometry provides 83 central contiguous difference-coarray lags and a one-arm aperture of 43d, where *d* is one-half wavelength. Under the normalized parametric coupling model adopted in this work, the reported numerical results show lower DOA-estimation RMSE than the compared L-shaped arrays over the investigated ranges. These results establish an analytical geometry and signal-model comparison rather than antenna-specific electromagnetic validation.

## 1. Introduction

Direction-of-arrival (DOA) estimation is a central problem in array signal processing. For two-dimensional estimation, an L-shaped array combines two orthogonal linear apertures and permits the two directional parameters to be processed separately or paired through cross-covariance information [1,2,3]. Its right-angle layout is compact relative to a filled planar array, although the suitability of any physical implementation still depends on the antenna element, platform, and required field of view.

Early L-shaped configurations commonly used two uniform linear arrays (ULAs), for which aperture and the number of resolvable sources scale directly with the number of physical sensors. Sparse-array design redistributes a fixed sensor budget to enlarge the physical aperture and generate a richer difference coarray. Foundational examples include minimum-redundancy, nested, and coprime arrays [4,5,6]. Super-nested and augmented-nested designs further show that rearranging close sensor pairs can preserve or enlarge the useful coarray while reducing geometry-level susceptibility to short-range mutual coupling [7,8,9]. Related developments include enhanced dilated and super augmented nested arrays [10,11], generalized nested-array families [12], and difference–sum coarray processing [13].

For L-shaped sparse processing, Elbir used two coprime branches and cross-covariance reconstruction to obtain automatically paired estimates [14]. Wu et al. proposed the L-shaped optimized interleaved array configuration (LOIAC), whose one-branch construction has a closed-form sensor placement and enlarged uniform DOF and aperture [15]. Peng et al. subsequently developed a different spatial–temporal processing route in which either L-shaped branch may be an arbitrary linear sparse array; the method constructs a virtual planar sparse array and applies two-dimensional spatial smoothing [16]. L-shaped processing has also been investigated for low-complexity underwater-robot DOA estimation [17]. These studies demonstrate that array geometry and the paired processing method are separate design choices.

Mutual coupling perturbs the nominal array manifold and can bias MUSIC-type estimates [18,19,20]. One response is to estimate, rectify, or compensate coupling and other receiver errors in the signal domain [19,20,21,22,23,24]. Florio et al. proposed an α-matrix-based linear calibration technique that captures systematic RF-chain amplitude/phase mismatches and a first-order approximation of mutual coupling in phase-interferometric AoA estimation [25]. This hardware-dependent signal-domain compensation is complementary to geometry-domain sparse-array design. A complementary geometry-domain response is to reduce the multiplicity of short physical separations, as in super-nested, thinned-coprime, augmented-nested, MISC, and related sparse arrays [7,9,26,27,28,29,30]. The present work follows the latter route and does not replace calibration when appreciable hardware errors remain. Third-order and higher-order sparse structures are already established in one-dimensional coarray processing [8,27,31]; therefore, the generic use of three subarrays is not claimed as the novelty.

To optimize the geometrical structure of L-shaped sparse arrays, improve the degrees of freedom, and reduce sensitivity to mutual coupling, this study proposes an L-shaped three-subarray sparse array for two-dimensional DOA estimation. The term “three-subarray” describes the physical branch construction and is distinct from the statistical order of a cumulant. The specific contribution is the closed-form admissible position family and its central contiguous coarray expression, together with cross-covariance-based paired 2D processing. The geometry is evaluated as a trade-off among contiguous uDOF, aperture, and sensitivity to the adopted distance-indexed coupling model, rather than as a universally least-coupled configuration. The main contributions of this study are summarized as follows:

1. A closed-form three-subarray branch construction is defined for N∈N with N≥4, T=3N, and M=⌈N/2⌉, and two identical branches are placed orthogonally with one shared sensor.
2. Based on the mathematical expression of the element configuration, the degrees-of-freedom expression of the proposed array is derived. The complete difference set, contiguous uDOF, smoothing dimension, and MUSIC source-count condition are distinguished explicitly.
3. Simulation experiments compare the 2D DOA estimation performance of common L-shaped sparse arrays and the proposed array, under the adopted normalized coupling model rather than antenna-specific electromagnetic validation.

The remainder of this paper is organized as follows. Section 2 analyzes the proposed geometry and its difference coarray. Section 3 presents the branch-separable signal model and the conditional two-stage estimator. Section 4 reports numerical results under the stated signal and coupling assumptions. Finally, Section 5 summarizes the findings, limitations, and directions for antenna-specific validation.

Table 1 summarizes the scope of representative designs and the proposed array.

Throughout this paper, the following notational conventions are adopted. Calligraphic uppercase letters denote sets, italic lowercase letters denote scalars, bold lowercase letters denote vectors, and bold uppercase letters denote matrices. The transpose, conjugate, and conjugate transpose operations are denoted by ${\left[\cdot \right]}^{T}$, ${\left[\cdot \right]}^{*}$, and ${\left[\cdot \right]}^{H}$, respectively. The mathematical expectation is represented by E(·). The vectorization operator is denoted by vec(·), and diag(·) denotes a diagonal matrix. The M×M identity matrix is denoted by $I_{M}$. The ceiling and floor operations are represented by ⌈·⌉ and ⌊·⌋, respectively. The Frobenius norm is denoted by ${∥\cdot ∥}_{F}$, and the Moore–Penrose pseudoinverse is denoted by ${\left[\cdot \right]}^{†}$. The symbols ⊙ and ⊗ denote the Khatri–Rao and Kronecker products, respectively.

## 2. Array Structure of the Proposed L-Shaped Sparse Array

Existing L-shaped sparse arrays emphasize different objectives. The LCA and LOIAC enlarge the virtual aperture and support paired estimation but do not explicitly optimize a distance-indexed coupling metric [14,15]; generalized L-shaped, tensor, and three-level structures provide other coarray and processing trade-offs [32,33,34,35]. This motivates a closed-form geometry whose contiguous coarray, aperture, and short-separation multiplicities can be evaluated together under a fixed physical-sensor budget.

This section defines the proposed geometry and derives the aperture, central contiguous coarray size, and short-separation pair multiplicities used in the subsequent comparison [14,15,32,33,34,35,36].

### 2.1. Mathematical Expression of Array Structure

The proposed L-shaped array consists of two mutually orthogonal branches with a common reference sensor at the origin. Each branch contains three sparse subarrays. Let d=λ/2 denote the location-grid spacing. The branch construction is illustrated in Figure 1 and is defined for N∈N with N≥4, T=3N, and M=⌈N/2⌉=⌊(N+1)/2⌋; consequently, each branch contains *T* distinct sensors and the complete L-shaped array contains Q=2T−1=6N−1 distinct physical sensors.

Each branch is composed of three disjoint subarrays. Subarray 1 contains N−1 sensors with spacing (N−1)d. Subarray 2 contains T−2N+1=N+1 sensors with spacing (2N−1)d, and Subarray 3 contains *N* sensors with spacing Nd. Subarray 1 supplies the short-lag basis; the spacing 2N−1 of Subarray 2 extends the covered lag families; and the spacing *N* and the offset of Subarray 3 bridge the remaining finite difference blocks. Consequently, each branch contains exactly T=3N distinct physical sensors. The bounded interval coverage is proven in Appendix A.

The normalized element positions are given below, where S, $S_{1}$, $S_{2}$, and $S_{3}$ denote one complete branch and its three constituent subarrays, respectively:

(1) $$\begin{array}{cc} S & =S_{1}\cup S_{2}\cup S_{3},\\ S_{1} & =\{\left(N-1\right)i_{1}|0\leq i_{1}\leq N-2\},\\ S_{2} & =\{\left(2N-1\right)i_{2}+M\left(N-1\right)|1\leq i_{2}\leq T-2N+1\},\\ S_{3} & =\{Ni_{3}+\left(2N-1\right)\left(T-2N\right)-{\left(N-1\right)}^{2}+2M\left(N-1\right)\\ \; & \;|0\leq i_{3}\leq N-1\}. \end{array}$$

The three position sets in (1) are pairwise disjoint for N≥4. They contain N−1, T−2N+1=N+1, and *N* sensors, respectively, and hence |S|=T=3N. The physical coordinate associated with normalized location $s_{i}\in S$ is $s_{i}d$. For the representative case N=4, T=12, and M=2, (1) gives S={0,3,6,13,20,27,31,34,35,39,41,43}, which is identical to the position vector used in the reported numerical results.

### 2.2. Degrees of Freedom and Array Aperture

For a normalized branch position set S, define the complete difference set as

(2) $$D=S-S=\{s_{m}-s_{n}|s_{m},s_{n}\in S\}.$$

The self-difference set of Subarray *i* is $D_{ii}=S_{i}-S_{i}$, and its cross-difference set with Subarray *j* is $D_{ij}=S_{i}-S_{j}$. Thus, $D={⋃}_{i=1}^{3}{⋃}_{j=1}^{3}D_{ij}$. Let $D_{c}=\left\{-L_{c},\ldots ,L_{c}\right\}$ denote the largest central contiguous subset of D. The full-lag DOF is $D_{\mathrm{tot}}=\left|D\right|$, whereas the contiguous uniform DOF used by the coarray processor is $D_{u}=\left|D_{c}\right|=2L_{c}+1$. Lags outside $D_{c}$ are not used by the present SS-MUSIC implementation.

The one-arm physical aperture is A=[max(S)−min(S)]d. A larger $D_{u}$ increases the available virtual dimension, and a larger aperture can improve angular resolution. Neither quantity alone guarantees lower finite-sample RMSE, which also depends on source separation, SNR, snapshots, estimator conditioning, and model mismatch.

For the admissible family, Appendix A proves that

(3) $$D_{u}=\left\{\begin{array}{cc} 2(2N-1)(T-2N+2)-1, & N\geq 4\;\mathrm{is}\;\mathrm{even},\\ 2(2N-1)(T-2N+2)+2N-3, & N\geq 5\;\mathrm{is}\;\mathrm{odd}. \end{array}\right.$$

For N=4, T=12, M=2, and Q=23, the one-arm aperture is 43d and $D_{c}=\left\{-41,\ldots ,41\right\}$, giving $D_{u}=83$. For the odd example N=5, T=15, and M=3, the branch is {0,4,8,12,21,30,39,48,53,57,58,63,66,68,73}; its central segment is {−66,…,66} and $D_{u}=133$.

Figure 2 illustrates the physical sensor positions and branch difference lags for the representative N=4 configuration.

## 3. Signal Model and Estimation Method

### 3.1. Signal Model

As shown in Figure 3, the proposed array has two mutually orthogonal branches along the *x*- and *z*-axes. Assume that there are *K* narrowband and mutually uncorrelated far-field sources impinging on the proposed L-shaped sparse array from *K* different directions. The array consists of *Q* physical elements. In this study, the number of sources is assumed to be known a priori. MDL or AIC may be used before the estimator [37]; source enumeration is not a contribution of the present geometry.

The incident signals are assumed to satisfy the following conditions:

1. All signal sources are in the far field of the proposed array.
2. All incident signals are narrowband signals, whose bandwidths are much smaller than their carrier frequencies.
3. The source signals are mutually uncorrelated, with $E\left\{s\left(t\right)s^{H}\left(t\right)\right\}=R_{s}$.
4. The branch noises are spatially and temporally white, mutually independent and independent of the sources.
5. The receiving elements have identical ideal patterns in the nominal signal model.

Let $\vartheta_{k}$ denote the conventional azimuth measured from the positive *x*-axis to the projection of the arrival direction on the xy-plane, and let $\varphi_{k}$ denote the elevation measured from the xy-plane toward the positive *z*-axis. The branch direction cosines are

(4) $$u_{x,k}=\cos\varphi_{k}\cos\vartheta_{k},\;u_{z,k}=\sin\varphi_{k}.$$

For one-dimensional branch processing, define the electrical angles $\mu_{k}=\arcsin\left(u_{x,k}\right)$ and $\nu_{k}=\arcsin\left(u_{z,k}\right)$. An electrical-angle pair corresponds to a physical three-dimensional arrival direction only if

(5) $$\sin^{2}\mu_{k}+\sin^{2}\nu_{k}\leq 1.$$

Within the selected unambiguous sector, the conventional angles can then be recovered as

(6) $$\varphi_{k}=\arcsin\left(\sin\nu_{k}\right),\;\vartheta_{k}=\arccos\;\left(\frac{\sin\mu_{k}}{\cos\varphi_{k}}\right).$$

The numerical section reports the two branch electrical-angle parameters used by the implemented phase model; arbitrary input pairs are not interpreted as conventional spherical azimuth/elevation pairs. This branch-projection parameterization follows the foundational L-shaped model [38] and its sparse extensions [14,15,32,39].

Ignoring mutual coupling initially, the received branch vectors are

(7) $$\begin{array}{cc} x(t) & =\sum_{k=1}^{K}a_{x}\left(\mu_{k}\right)s_{k}\left(t\right)+n_{x}\left(t\right),\\ z(t) & =\sum_{k=1}^{K}a_{z}\left(\nu_{k}\right)s_{k}\left(t\right)+n_{z}\left(t\right). \end{array}$$

Equivalently,

(8) $$\begin{array}{cc} x(t) & =A_{x}s\left(t\right)+n_{x}\left(t\right),\\ z(t) & =A_{z}s\left(t\right)+n_{z}\left(t\right), \end{array}$$

where $s\left(t\right)={\left[s_{1}\left(t\right),\ldots ,s_{K}\left(t\right)\right]}^{T}$, $A_{x}=\left[a_{x}\left(\mu_{1}\right),\ldots ,a_{x}\left(\mu_{K}\right)\right]\in C^{T\times K}$, and $A_{z}=\left[a_{z}\left(\nu_{1}\right),\ldots ,a_{z}\left(\nu_{K}\right)\right]\in C^{T\times K}$. If $\left(p_{x,w},0,0\right)$ and $\left(0,0,p_{z,w}\right)$ are the physical coordinates of the *w*th sensors on the x and z branches, respectively, then

(9) $$\begin{array}{cc} {\left[a_{x}\left(\mu_{k}\right)\right]}_{w} & =\exp\left(-j\frac{2\pi }{\lambda }p_{x,w}\sin\mu_{k}\right),\\ {}[a_{z}\left(\nu_{k}\right){]}_{w} & =\exp\left(-j\frac{2\pi }{\lambda }p_{z,w}\sin\nu_{k}\right). \end{array}$$

With within-branch coupling, the adopted normalized signal-domain model is

(10) $$\begin{array}{cc} x(t) & =C_{x}A_{x}s\left(t\right)+n_{x}\left(t\right),\\ z(t) & =C_{z}A_{z}s\left(t\right)+n_{z}\left(t\right). \end{array}$$

Let $S_{\xi }=\left\{s_{\xi ,1},\ldots ,s_{\xi ,T}\right\}\subset Z$ denote the normalized positions on branch ξ∈{x,z}. Following sparse-array coupling models whose entries are indexed by physical grid separation [27,30], define

(11) $${\left[C_{\xi }\right]}_{m,n}=\left\{\begin{array}{cc} c_{|s_{\xi ,m}-s_{\xi ,n}|}, & |s_{\xi ,m}-s_{\xi ,n}|\leq B,\\ 0, & |s_{\xi ,m}-s_{\xi ,n}|>B, \end{array}\right.\;m,n=1,\ldots ,T.$$

Here $c_{0}=1>\left|c_{1}\right|>\ldots >\left|c_{B}\right|>0$. A representative distance-decaying sequence used in sparse-array studies is given by [27,29]:

(12) $$c_{1}=\rho e^{j\pi /3},\;c_{b}=\frac{c_{1}}{b}e^{-j(b-1)\pi /8},\;b=2,\ldots ,B$$

Thus, a matrix entry is indexed by physical grid separation rather than sensor order. For a nonuniform branch, $C_{\xi }$ is symmetric but is generally not Toeplitz with respect to the matrix indices. The coefficients are normalized scenario parameters, not measured S-parameters of a specified antenna.

For a normalized branch set S, define the weight function and normalized coupling leakage as follows [27,28,29,30]:

(13) $$\begin{array}{cc} w(\ell ) & =\left|\{\left(s_{m},s_{n}\right)\in S^{2}:s_{m}-s_{n}=\ell \}\right|,\\ L_{\mathrm{mc}} & =\frac{{∥C-\mathrm{diag}\left(C\right)∥}_{F}}{{∥C∥}_{F}}. \end{array}$$

Under the *B*-truncated coefficient model, w(1),…,w(B) count the positively oriented sensor pairs associated with nonzero short-range coupling orders. Since w(−b)=w(b),

(14) $$L_{\mathrm{mc}}^{2}=\frac{2\sum_{b=1}^{B}w\left(b\right){\left|c_{b}\right|}^{2}}{T+2\sum_{b=1}^{B}w\left(b\right){\left|c_{b}\right|}^{2}}.$$

Smaller critical weights and smaller $L_{\mathrm{mc}}$ indicate lower geometry/model-level susceptibility under the same coefficient family. In this paper, “mutual-coupling-resistant” means reduced geometry-level sensitivity assessed by the short-separation weights, normalized leakage, and the observed first-stage degradation as $|c_{1}|$ increases; it does not denote an antenna-independent electromagnetic guarantee. Neither quantity alone determines measured S-parameters, spectral-peak displacement, mainlobe width, or RMSE.

The two branches have identical normalized position sets after a 90° rotation, so a fully coupled nominal two-branch model would use the same within-branch coefficient law for $C_{x}$ and $C_{z}$. The symbols are retained separately because fabrication and platform asymmetries can make physical matrices unequal. The present model neglects cross-branch electromagnetic coupling and retains the standard branch-separable L-shaped model [14,15,32,34,38]. Cross-branch coupling is antenna- and platform-dependent and lies outside this geometry-level analysis.

In the supplied numerical implementation, the position-dependent coupling matrix is applied to the first-stage x-branch data. The conditional second-stage cross-covariance is generated from ideal branch manifolds using the first-stage estimates. Hence, the reported coupling-dependent curves quantify first-stage manifold mismatch and its propagation through the sequential estimator; they do not represent simultaneous coupling on both physical branches.

### 3.2. X-Branch Electrical-Angle Estimation

Let $R_{s}=\mathrm{diag}\left(\sigma_{1}^{2},\ldots ,\sigma_{K}^{2}\right)$ and $p={\left[\sigma_{1}^{2},\ldots ,\sigma_{K}^{2}\right]}^{T}$. For the nominal uncoupled processing model,

(15) $$\begin{array}{cc} R_{x}^{\left(0\right)} & =A_{x}R_{s}A_{x}^{H}+\sigma_{n}^{2}I_{T},\\ R_{z}^{\left(0\right)} & =A_{z}R_{s}A_{z}^{H}+\sigma_{n}^{2}I_{T}. \end{array}$$

For data generated with coupling, the actual branch covariances are

(16) $$\begin{array}{cc} R_{x}^{\left(c\right)} & =C_{x}A_{x}R_{s}A_{x}^{H}C_{x}^{H}+\sigma_{n}^{2}I_{T},\\ R_{z}^{\left(c\right)} & =C_{z}A_{z}R_{s}A_{z}^{H}C_{z}^{H}+\sigma_{n}^{2}I_{T}. \end{array}$$

Vectorizing the nominal covariances gives

(17) $$\begin{array}{cc} z_{x}^{\left(0\right)} & =\mathrm{vec}\left(R_{x}^{\left(0\right)}\right)=\left(A_{x}^{*}⊙A_{x}\right)p+\sigma_{n}^{2}\mathrm{vec}\left(I_{T}\right),\\ z_{z}^{\left(0\right)} & =\mathrm{vec}\left(R_{z}^{\left(0\right)}\right)=\left(A_{z}^{*}⊙A_{z}\right)p+\sigma_{n}^{2}\mathrm{vec}\left(I_{T}\right). \end{array}$$

For example, $A_{x}^{*}⊙A_{x}=\left[a_{x}^{*}\left(\mu_{1}\right)⊗a_{x}\left(\mu_{1}\right),\ldots ,a_{x}^{*}\left(\mu_{K}\right)⊗a_{x}\left(\mu_{K}\right)\right]$. The corresponding coupled-data vector is

(18) $$z_{x}^{\left(c\right)}=\left[{\left(C_{x}A_{x}\right)}^{*}⊙\left(C_{x}A_{x}\right)\right]p+\sigma_{n}^{2}\mathrm{vec}\left(I_{T}\right),$$

The same expression applies to the z branch. The estimator uses the nominal virtual manifold, so cases with $C_{\xi }\neq I_{T}$ are controlled uncalibrated model-mismatch tests rather than coupling-calibration experiments.

After equal-lag entries are combined and ordered, retain only the central contiguous set $D_{c}=\left\{-L_{c},\ldots ,L_{c}\right\}$. Denote the resulting vector by ${\bar{z}}_{x}\in C^{2L_{c}+1}$ and set $L_{s}=L_{c}+1$. Standard forward spatial smoothing [40,41] uses $L_{s}$ overlapping virtual subarrays of length $L_{s}$:

(19) $$\begin{array}{cc} z_{x,\ell } & ={\left[{\bar{z}}_{x}\left(\ell \right),{\bar{z}}_{x}\left(\ell +1\right),\ldots ,{\bar{z}}_{x}\left(\ell +L_{s}-1\right)\right]}^{T},\\ R_{\mathrm{ss},x} & =\frac{1}{L_{s}}\sum_{\ell =1}^{L_{s}}z_{x,\ell }z_{x,\ell }^{H}. \end{array}$$

Let $U_{n,x}\in C^{L_{s}\times \left(L_{s}-K\right)}$ be the noise-subspace eigenvector matrix of $R_{\mathrm{ss},x}$. The dimensionally compatible virtual-ULA steering vector and spectrum are

(20) $$\begin{array}{cc} {\tilde{a}}_{x}\left(\mu \right) & ={\left[1,e^{-j\pi \sin\mu },\ldots ,e^{-j\pi (L_{s}-1)\sin\mu }\right]}^{T}\in C^{L_{s}},\\ P_{x}\left(\mu \right) & =\frac{1}{{\tilde{a}}_{x}^{H}\left(\mu \right)U_{n,x}U_{n,x}^{H}{\tilde{a}}_{x}\left(\mu \right)}. \end{array}$$

Under the required rank and sample conditions, this smoothing construction supports at most $L_{s}-1$ uncorrelated sources. Thus, $D_{\mathrm{tot}}$, $D_{u}$, $L_{s}$, and the practically resolvable source count are related but not interchangeable.

### 3.3. Conditional Z-Branch Estimation and Pairing

Let *J* denote the number of snapshots and let ${\hat{\mu }}_{1},\ldots ,{\hat{\mu }}_{K}$ be the ordered first-stage estimates. Form

(21) $$\begin{array}{cc} {\hat{A}}_{x} & =[a_{x}\left({\hat{\mu }}_{1}\right),\ldots ,a_{x}\left({\hat{\mu }}_{K}\right)],\\ {\hat{R}}_{x} & =\frac{1}{J}\sum_{t=1}^{J}x\left(t\right)x^{H}\left(t\right),\;{\hat{R}}_{xz}=\frac{1}{J}\sum_{t=1}^{J}x\left(t\right)z^{H}\left(t\right). \end{array}$$

Under the nominal model,

(22) $$R_{xz}^{\left(0\right)}=A_{x}R_{s}A_{z}^{H}=\sum_{k=1}^{K}\sigma_{k}^{2}a_{x}\left(\mu_{k}\right)a_{z}^{H}\left(\nu_{k}\right).$$

Both branch manifolds multiply the same ordered source vector, and the *k*th factors therefore correspond to the same source when $R_{s}$ is diagonal. With coupling, $R_{xz}^{\left(c\right)}=C_{x}A_{x}R_{s}A_{z}^{H}C_{z}^{H}$, which introduces the same uncalibrated mismatch discussed above.

For the paired second stage, assume K<T, $\mathrm{rank}({\hat{A}}_{x})=K$, and an invertible source covariance estimate. If $\lambda_{1}\geq \ldots \geq \lambda_{T}$ are the eigenvalues of ${\hat{R}}_{x}$, estimate the physical-branch noise variance by

(23) $${\hat{\sigma }}_{n}^{2}=\frac{1}{T-K}\sum_{i=K+1}^{T}\lambda_{i}.$$

The source covariance and ordered z-branch manifold are then estimated as

(24) $$\begin{array}{cc} {\hat{R}}_{s} & ={\hat{A}}_{x}^{†}\left({\hat{R}}_{x}-{\hat{\sigma }}_{n}^{2}I_{T}\right){\left({\hat{A}}_{x}^{†}\right)}^{H},\\ {\hat{A}}_{z} & ={\left({\hat{R}}_{s}^{-1}{\hat{A}}_{x}^{†}{\hat{R}}_{xz}\right)}^{H}. \end{array}$$

The order of the factors in the second equation follows directly from $A_{x}^{†}R_{xz}=R_{s}A_{z}^{H}$ and is dimensionally consistent. After fixing the column order of ${\hat{A}}_{x}$, the least-squares reconstruction inherits that order subject to distinct sources, sufficient rank, and accurate first-stage estimates [3,14,42]. This is a conditional common-order property, not an unconditional finite-sample guarantee.

Let ${\hat{a}}_{z,k}$ denote the *k*th column of ${\hat{A}}_{z}$. Consistently with the supplied implementation, the source-conditioned rank-one matrix used for the paired second-branch spectrum is

(25) $${\hat{R}}_{z,k}={\hat{a}}_{z,k}{\hat{a}}_{z,k}^{H},\;k=1,\ldots ,K.$$

This is a model-based per-source construction, not the full finite-snapshot sample covariance $J^{-1}\sum_{t}z\left(t\right)z^{H}\left(t\right)$. Adding ${\hat{\sigma }}_{n}^{2}I_{T}$ would shift its eigenvalues without changing its eigenvectors and is therefore unnecessary for the implemented rank-one spectral search. Let $U_{n,z,k}$ contain the T−1 noise-subspace eigenvectors of ${\hat{R}}_{z,k}$. The paired spectrum is

(26) $$P_{z}\left(\nu |{\hat{\mu }}_{k}\right)=\frac{1}{a_{z}^{H}\left(\nu \right)U_{n,z,k}U_{n,z,k}^{H}a_{z}\left(\nu \right)}.$$

Because the estimator is sequential, errors in ${\hat{\mu }}_{k}$ perturb ${\hat{A}}_{x}$ and propagate to ${\hat{A}}_{z}$. The reported two-dimensional RMSE evaluates the complete two-stage procedure and therefore includes this effect. As in related L-shaped estimators [14,39], pairing and elevation accuracy can deteriorate at low SNR, for closely spaced sources, or under manifold mismatch.

## 4. Numerical Results

The following results are numerical evaluations under the narrowband far-field model, mutually uncorrelated sources, AWGN, a known or correctly supplied source number, ideal identical element responses, and the normalized coupling model defined in Section 3. In the supplied implementation, the coupling matrix is applied to the first-stage branch; the conditional second stage propagates the first-stage estimation error but does not include an independently coupled second branch. Every comparison array uses the same total number of physical sensors and the same first-stage signal, noise, coefficient, snapshot, and nominal uncalibrated SS-MUSIC settings. These simulations evaluate analytical geometry and relative signal-processing sensitivity under the adopted model; they do not constitute full-wave or measured validation of a particular antenna implementation.

The reported uDOF is a geometric quantity determined only by the sensor-position difference set. A common element pattern may change angle-dependent gain, effective SNR, and observability near a pattern null, but it does not delete geometric lags. Nonidentical embedded element patterns, pattern roll-off, and pattern-null effects are not included in the present ideal-element model.

The plotted angular coordinates are the branch electrical angles (μ,ν) defined in Section 3. Let $N_{\mathrm{MC}}$ denote the number of Monte Carlo trials. The reported two-branch RMSE is

(27) $$\mathrm{RMSE}=\sqrt{\frac{1}{N_{\mathrm{MC}}K}\sum_{r=1}^{N_{\mathrm{MC}}}\sum_{k=1}^{K}\left[{\left({\hat{\mu }}_{k,r}-\mu_{k}\right)}^{2}+{\left({\hat{\nu }}_{k,r}-\nu_{k}\right)}^{2}\right]}.$$

The SNR for source *k* is

(28) $$\mathrm{SNR}=10\log_{10}\left(\frac{P_{k}}{\sigma_{n}^{2}}\right),$$

where all sources use the same power $P_{k}$ and every sensor has noise variance $\sigma_{n}^{2}$.

All arrays are compared with the same total number of Q=23 physical sensors, fixing the number of receiving channels and the principal hardware-count constraint. Their aperture, footprint, difference-coarray size, and short-pair multiplicities are geometry-dependent outcomes and are intentionally not equalized. The comparison therefore addresses overall performance under equal sensor count; it does not isolate the causal contribution of aperture alone. Table 2 lists the one-branch position sets and resulting analytical quantities [5,6,14,15,16,33].

Figure 4 compares the deterministic central contiguous uDOF under increasing sensor counts. For the proposed geometry, the corrected values at N=7 (Q=41) and N=9 (Q=53) are 245 and 389, respectively. The boundary cases N=2,3 are omitted because the closed-form proposition applies from N=4.

Table 3 reports deterministic matrix quantities computed from the position sets in Table 2 using B=100 and $c_{b}=c_{1}e^{-j(b-1)\pi /8}/b$. LOIAC has the smallest leakage, whereas the proposed geometry provides the largest aperture and central uDOF under the same number of physical sensors. This is a design trade-off and does not establish a one-to-one mapping from $L_{\mathrm{mc}}$ to spectral-peak shift, mainlobe width, sidelobe level, or RMSE.

### 4.1. Simulation 1: Normalized Spatial Spectrum of the Proposed Array

This simulation illustrates the two-stage spectrum and the source-conditioned pairing procedure. The number of snapshots is set to 100 and the signal-to-noise ratio (SNR) is fixed at 0 dB. Three test directions are applied successively at $\left(\mu_{1},\nu_{1}\right)=\left(-65{}^{\circ},50{}^{\circ}\right)$, $\left(\mu_{2},\nu_{2}\right)=\left(35{}^{\circ},20{}^{\circ}\right)$, and $\left(\mu_{3},\nu_{3}\right)=\left(70{}^{\circ},70{}^{\circ}\right)$.

Figure 5a shows peaks at the three x-branch electrical angles, and Figure 5b–d shows the conditional z-branch spectra generated after inserting the corresponding first-stage estimates. Because the cases are applied successively, this example illustrates the common-column-order mechanism in Section 3.3 rather than proving simultaneous multisource pairing at finite sample size.

### 4.2. Simulation 2: Comparison of Short-Separation Weight Functions

For Q=23, Figure 6 compares the branch weight functions of the four geometries in Table 2.

The critical values are [w(1),w(2),w(3)]=[11,10,9] for the ULA, [2,2,2] for the LCA, [1,1,1] for LOIAC, and [1,2,3] for the proposed array. Under a common distance-decaying coefficient family, the ULA has the largest short-range off-diagonal coupling energy, while LOIAC has the smallest critical close-pair multiplicity. The proposed array provides a different compromise, with $D_{u}=83$ and aperture 43d compared with $D_{u}=65$ and aperture 39d for LOIAC. The weight function and $L_{\mathrm{mc}}$ are geometry/model-level indicators; final RMSE also depends on aperture, virtual-array dimension, source scene, SNR, snapshots, and manifold mismatch.

Mutual coupling does not change physical coordinates and therefore cannot shorten the geometric set $D_{c}$. Increasing the coupling order or magnitude instead increases covariance/manifold mismatch and can reduce the usable estimation performance of the nominal coarray processor.

### 4.3. Simulation 3: X-Branch Estimation Under High Source Counts

This subsection evaluates only the resolving capability of the first-branch coarray SS-MUSIC processor. It does not invoke the physical cross-covariance reconstruction of the paired second branch, whose present closed-form solution requires K<T and the stated full-rank conditions. The purpose of this simulation is to investigate the capability of different L-shaped arrays in high source-count and underdetermined first-branch estimation. Since the element positions along the *x*-axis and *z*-axis are identical, only the x-branch electrical-angle spectrum is evaluated. In general, a relatively large number of snapshots is required for multisource estimation. Accordingly, in this experiment, the number of snapshots is increased to 1000, the signal-to-noise ratio (SNR) is set to 0 dB, and the normalized nearest-grid coupling magnitude is set to $|c_{1}|=0.1$. To ensure fairness in comparison and maintain effective control of variables, all arrays are configured with 23 physical elements.

The 12-sensor ULA branch is evaluated with 11 sources, its nominal MUSIC limit, uniformly spaced from −50° to 50° (Figure 7a). The LCA, LOIAC, and proposed sparse branches are each evaluated with 20 sources (Figure 7b–d). These panels demonstrate resolvable peaks in the respective high source-count settings. Because the ULA panel uses a different number of sources, Figure 7 is not used as a same-task RMSE ranking. Moreover, this one-branch experiment does not test the conditional second-stage pairing requirement K<T.

### 4.4. Simulation 4: RMSE Versus SNR at Different Normalized Coupling Levels

This experiment compares the four arrays as the SNR varies under three normalized coupling settings. The number of Monte Carlo trials is 200, the number of snapshots is fixed at 100, and the signal-to-noise ratio (SNR) increases from −15 dB to 15 dB with an interval of 3 dB. Figure 8a–c uses $|c_{1}|=0.1$, 0.3, and 0.5, respectively. These values are normalized nearest-grid coupling settings in the adopted coefficient family, not universal conversions to a measured spacing or S-parameter.

All arrays are processed with the same nominal steering model and without estimating the first-stage coupling matrix $C_{x}$. The curves therefore include first-stage coupling-induced manifold mismatch and its propagation into the conditional second stage. RMSE decreases with SNR in the reported cases, and the proposed geometry gives the lowest plotted RMSE across the investigated ranges. This end-to-end result reflects the combined effects of its 43d aperture, $D_{u}=83$ central coarray, short-pair distribution, source scene, and estimator conditioning. It must not be attributed to uDOF or $L_{\mathrm{mc}}$ alone and does not establish simultaneous two-branch coupling robustness.

### 4.5. Simulation 5: RMSE Versus Snapshots at Different Normalized Coupling Levels

This experiment compares the same four arrays as the number of snapshots varies under three normalized coupling settings. The number of Monte Carlo trials is set to 200, the SNR is fixed at −6 dB, and the number of snapshots increases from 100 to 1000 with an interval of 100. Figure 9a–c again uses $|c_{1}|=0.1$, 0.3, and 0.5 as normalized coupling-severity settings.

RMSE decreases as more snapshots are used in the reported cases. The proposed array has the lowest plotted RMSE over the investigated snapshot and coupling ranges, but the curves do not isolate a single causal factor: aperture, central virtual dimension, short-separation multiplicity, finite-sample covariance error, sequential-estimation error, and uncalibrated coupling mismatch all contribute. Accordingly, the result is an equal-sensor-count end-to-end comparison under the adopted signal model, not a universal electromagnetic robustness claim.

## 5. Conclusions

This paper presented a closed-form three-subarray branch construction and its orthogonal L-shaped extension for 2D DOA estimation. A distinctive feature of the proposed array is its specific disjoint three-subarray position family, rather than the generic use of three subarrays. For the admissible family N∈N with N≥4, T=3N, and M=⌈N/2⌉, the geometry has Q=6N−1 distinct physical sensors; the representative Q=23 configuration provides a one-arm aperture of 43d and 83 central contiguous coarray lags.

The proposed geometry provides a large contiguous coarray and aperture while limiting the number of short-separation pairs. These quantities form a trade-off rather than independent guarantees of lower RMSE; estimation error also depends on source separation, SNR, snapshots, estimator conditioning, and manifold mismatch.

In terms of mathematical formulation, a complete mathematical expression system is established for the proposed array. The revised formulation distinguishes physical angles from branch electrical angles, uses dimensionally matched virtual steering vectors, and states the conditions required by cross-covariance pairing. The coupling matrix is indexed by normalized physical separation rather than sensor order.

Under equal sensor count and the investigated SNR, snapshot, and normalized coupling ranges, the proposed geometry gives lower plotted RMSE than the compared arrays. LOIAC has the lowest critical close-pair multiplicity and normalized leakage, so the proposed array is not claimed to be universally the least-coupled geometry. The reported comparison instead documents the trade-off among aperture, central uDOF, close-pair multiplicity, normalized leakage, and end-to-end estimation error under the stated model.

The present validation is limited to analytical coarray results and a narrowband parametric signal-domain coupling model. In the supplied implementation, coupling is applied to the first-stage branch and its estimation error propagates to the conditional second stage; simultaneous coupling on both branches is not evaluated. At the antenna-implementation level, array behavior depends on radiator geometry, material stack-up, inter-element spacing, and operating frequency. For example, Citroni and Livreri optimized a 1×2 bowtie linear array for mid-infrared energy harvesting [43]. Although that antenna-specific study addresses a different objective from the present difference-coarray/DOA design, it illustrates why full-wave validation must be tied to a specified element and frequency band. Full-wave or S-parameter simulation, embedded and nonidentical element patterns, cross-branch electromagnetic coupling, feed-network and platform effects, calibration errors, and prototype measurements require an antenna-specific study and remain future work.

## Figures and Tables

**Figure 1:**
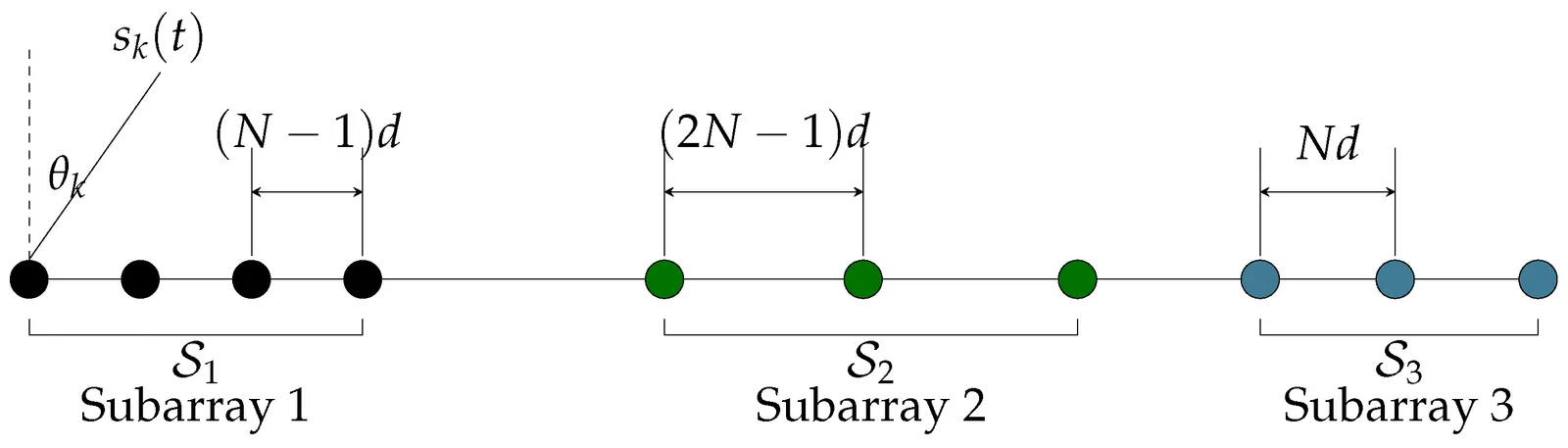
Three-subarray branch schematic (not to scale). Black, green and teal sensors denote Subarrays 1, 2 and 3; double-headed arrows indicate within-subarray spacings, and the oblique line indicates an incident direction relative to the branch broadside. Here d=λ/2.

**Figure 2:**
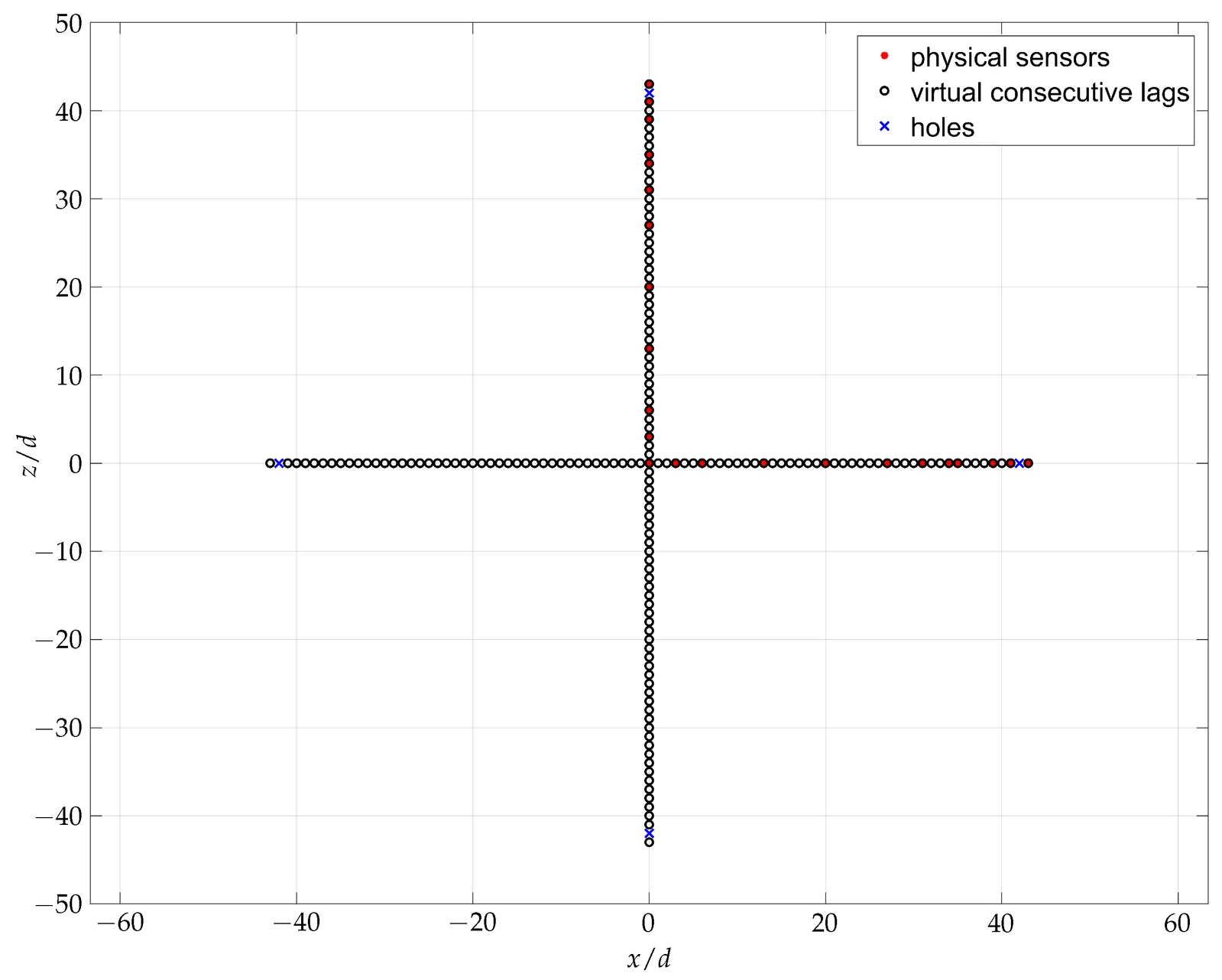
Physical sensors and central contiguous virtual lags of the proposed L-shaped geometry. Both axes show normalized position in units of d=λ/2.

**Figure 3:**
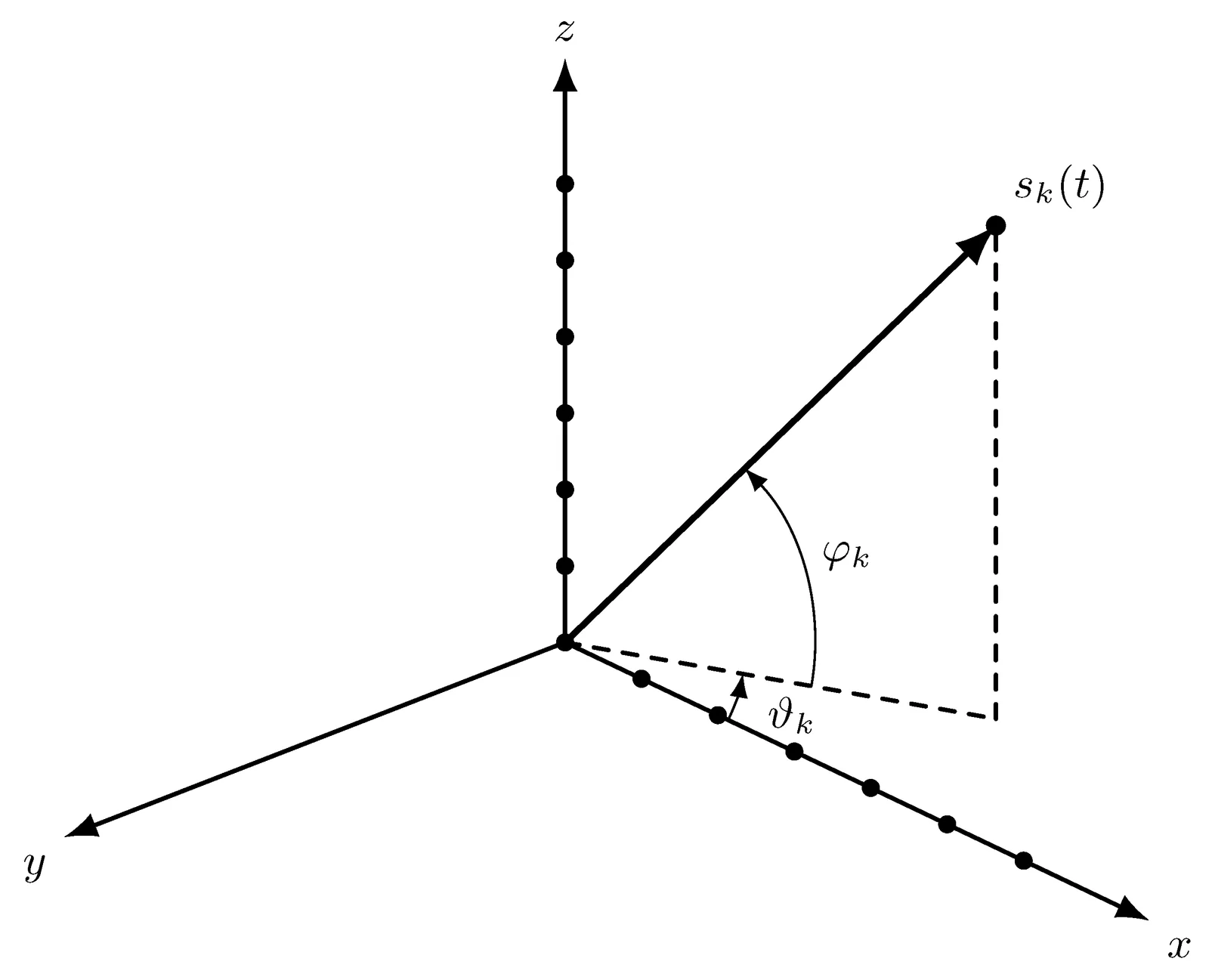
Coordinate convention for azimuth $\vartheta_{k}$ and elevation $\varphi_{k}$.

**Figure 4:**
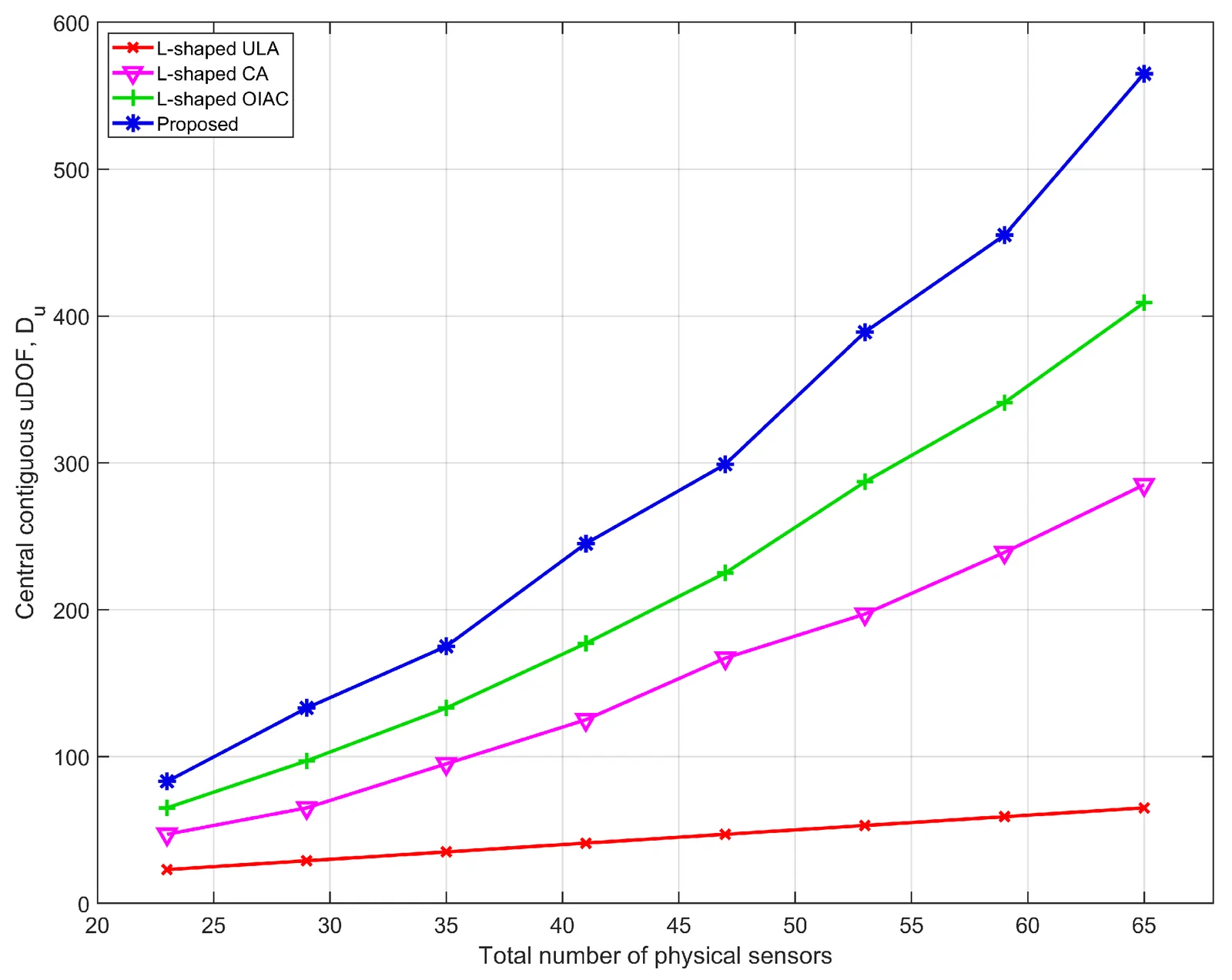
Number of central contiguous difference-coarray lags versus the total number of physical sensors. All curves are deterministic geometry results. The proposed-array curve is shown for its admissible range N≥4, where Q=6N−1 and M=⌈N/2⌉.

**Figure 5:**
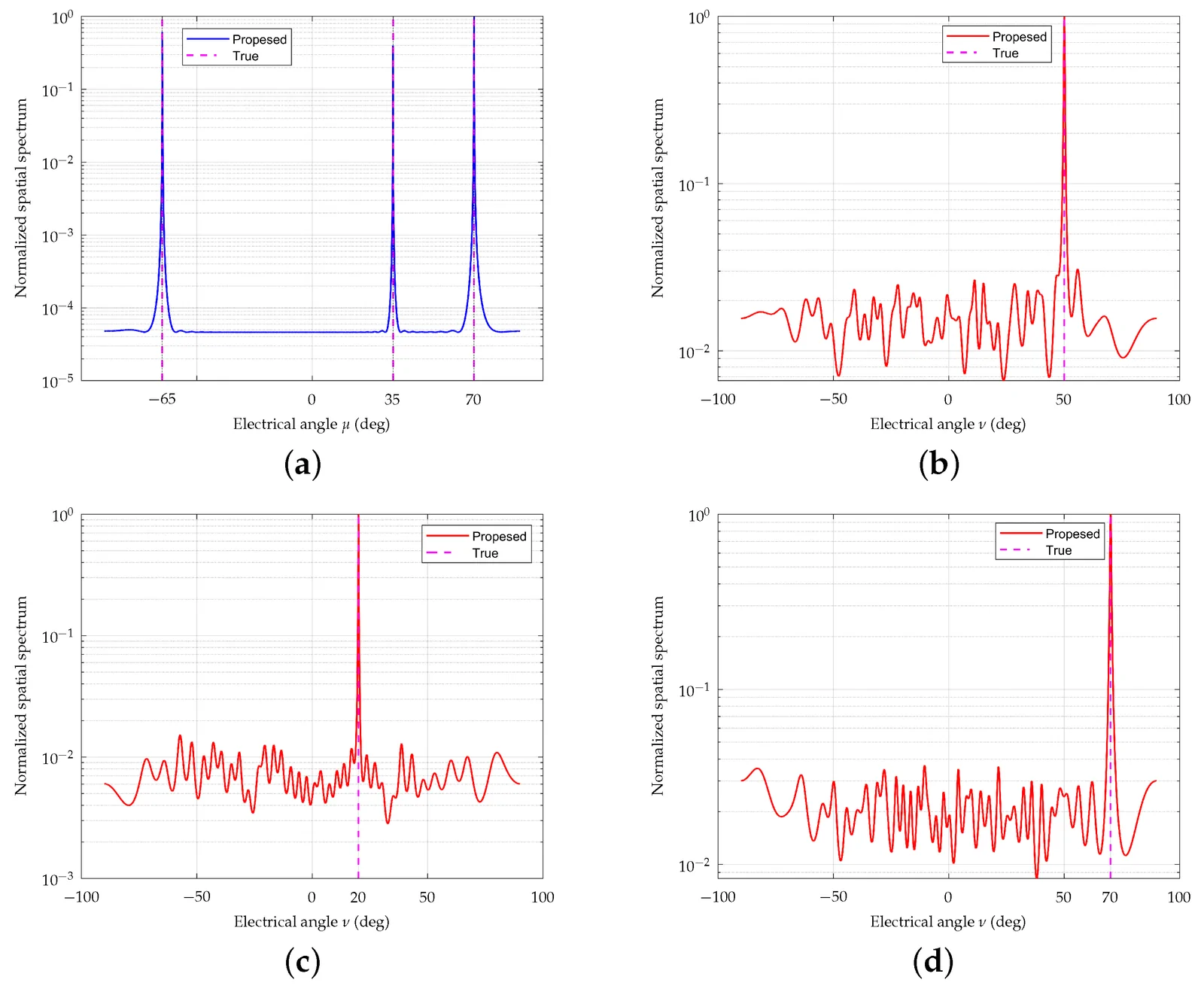
Normalized electrical-angle spectra of the proposed array. (**a**) Normalized x-branch electrical-angle spectrum. (**b**) $\left(\mu_{1},\nu_{1}\right)=\left(-65{}^{\circ},50{}^{\circ}\right)$. (**c**) $\left(\mu_{2},\nu_{2}\right)=\left(35{}^{\circ},20{}^{\circ}\right)$. (**d**) $\left(\mu_{3},\nu_{3}\right)=\left(70{}^{\circ},70{}^{\circ}\right)$.

**Figure 6:**
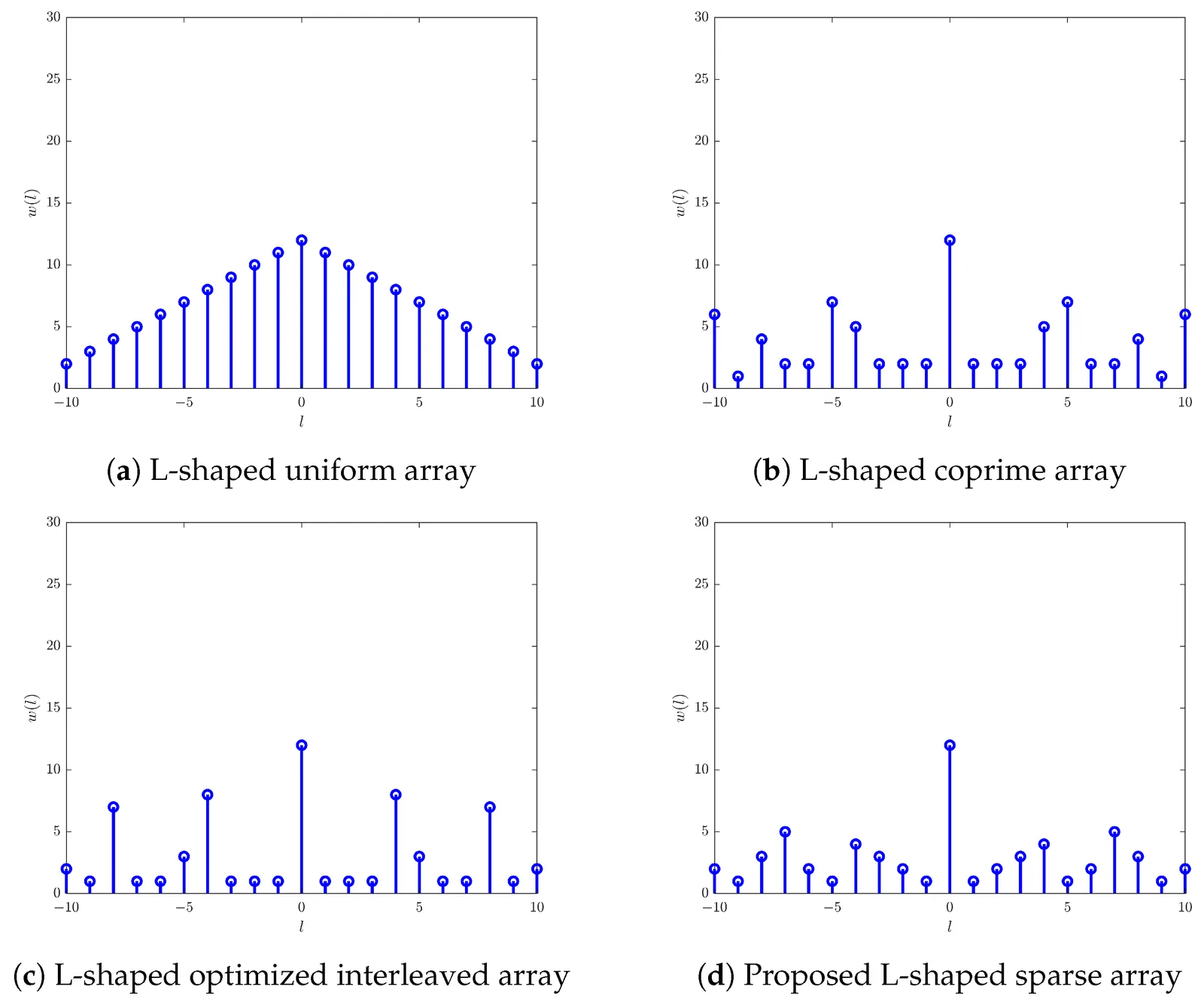
Branch weight functions of four L-shaped arrays with 23 physical sensors.

**Figure 7:**
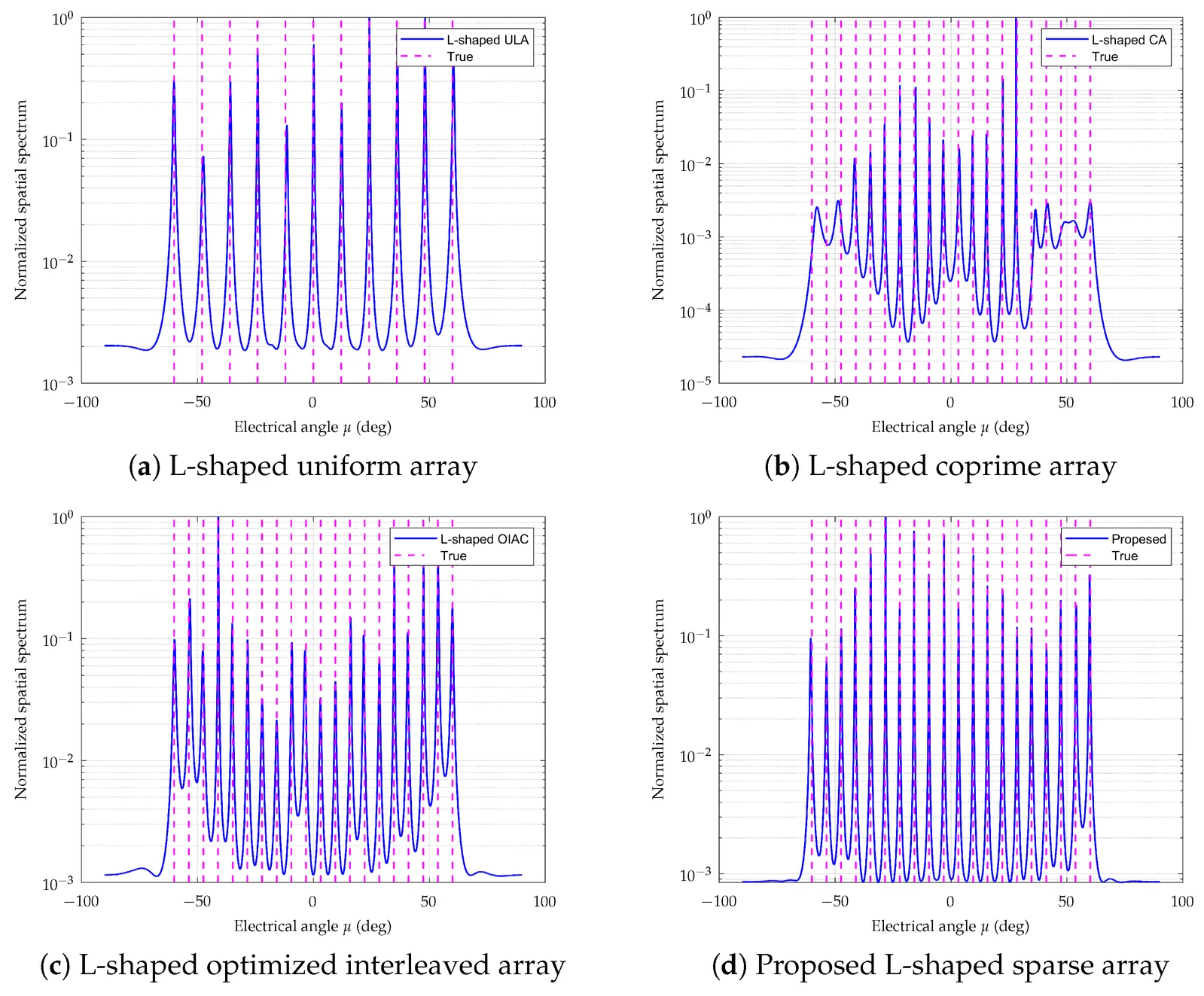
Normalized x-branch electrical-angle spectra at high source counts.

**Figure 8:**
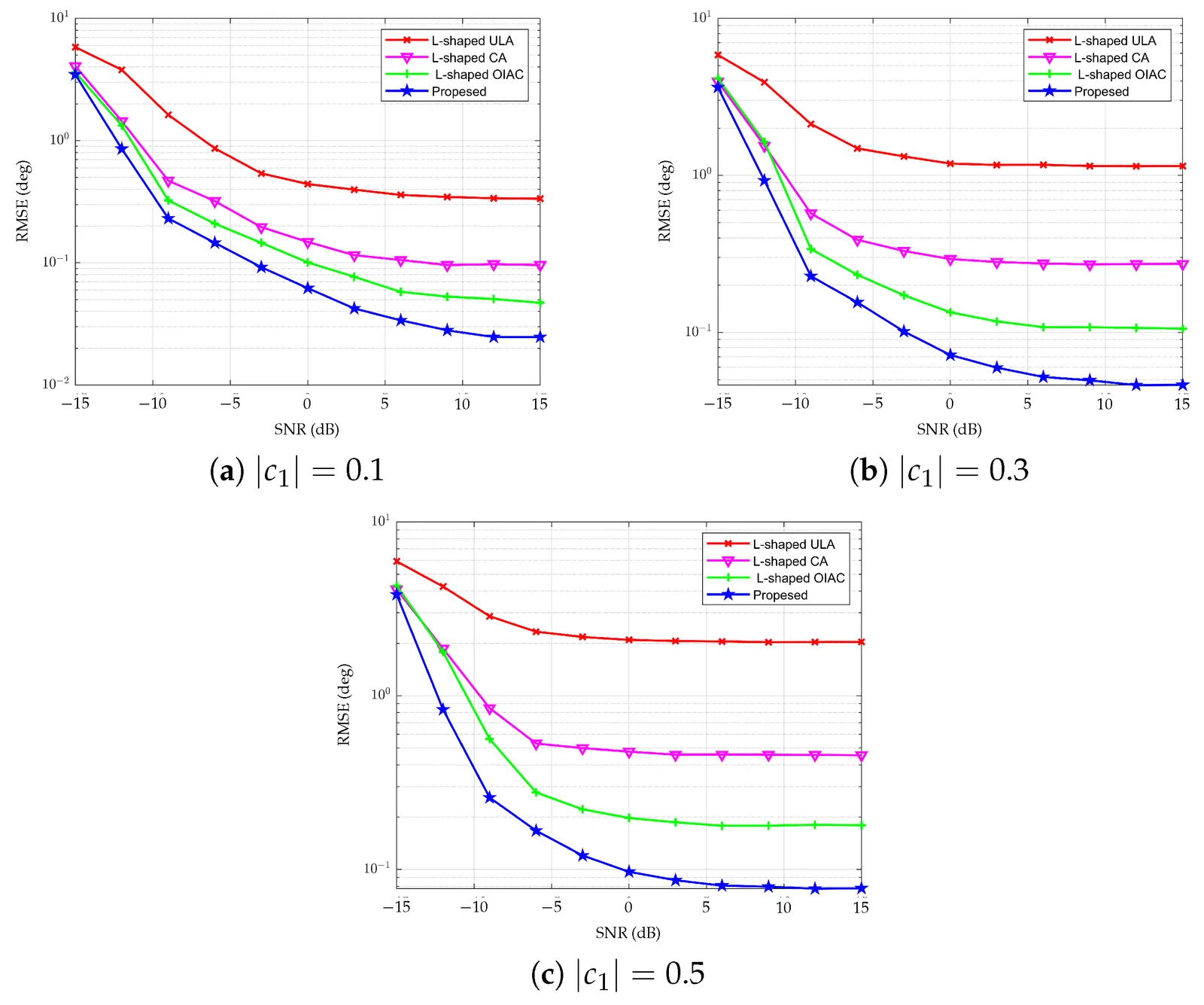
RMSE versus SNR at three normalized coupling levels.

**Figure 9:**
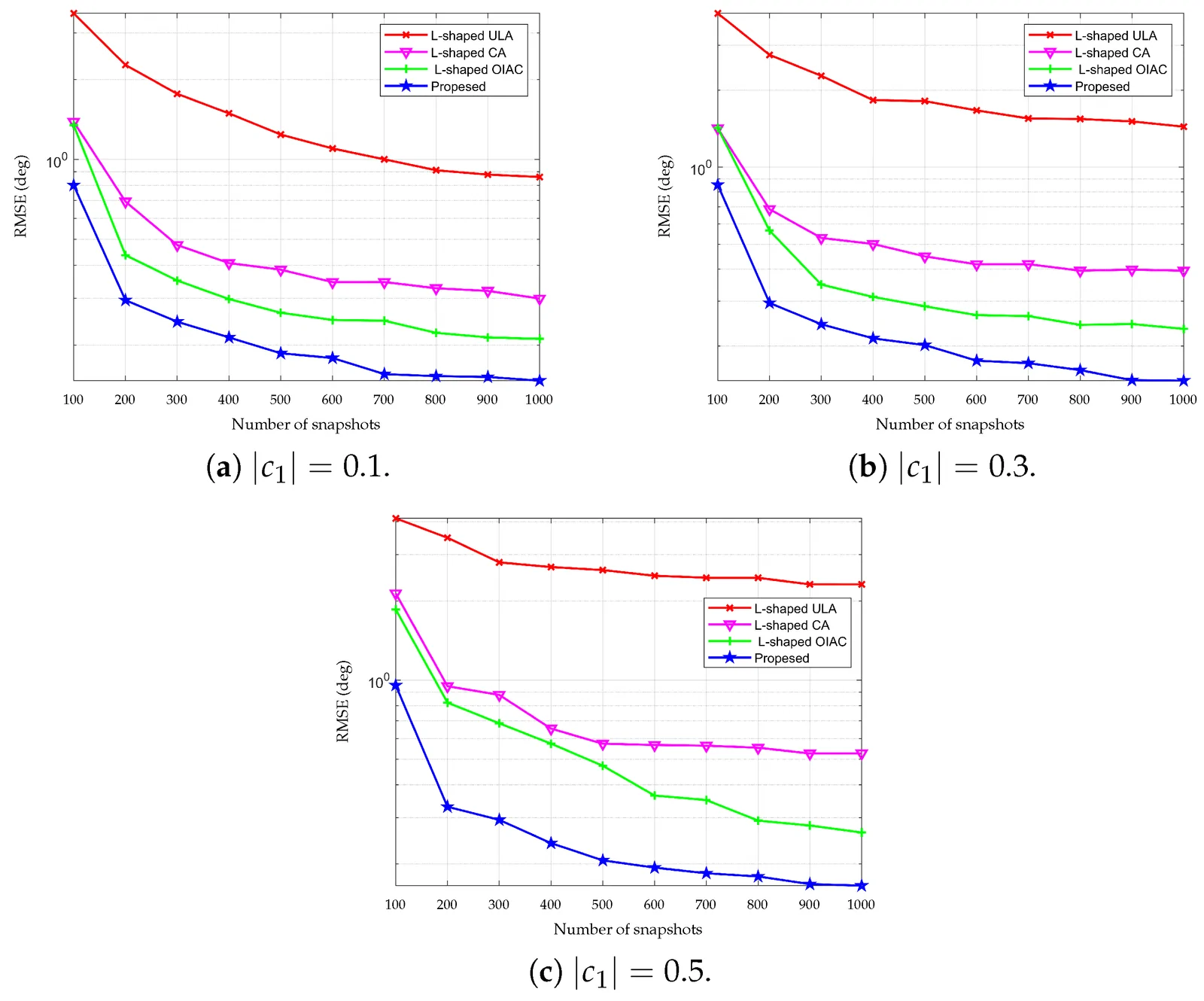
RMSE versus the number of snapshots at three normalized coupling levels.

**Table 1:** Scope comparison with representative high-order and L-shaped sparse-array designs.

Design	Branch Construction	Analysis Domain	2D Pairing	Coupling Treatment
L-shaped coprime array [14]	Two coprime linear branches	One-dimensional second-order coarrays	Cross-covariance pairing	No explicit coupling model
LOIAC [15]	Two optimized interleaved branches	One-dimensional second-order coarrays	Cross-covariance pairing	No explicit coupling model
Generic L-shaped sparse processing [16]	Two arbitrary sparse linear branches	Spatial–temporal virtual planar coarray	2D spatial smoothing	No explicit coupling model
Third-order nested array [31]	Closed-form linear sparse geometry	Third-order exhaustive coarray/cumulants	Not an L-shaped 2D method	Not its primary criterion
MISC array [27]	Three sparse ULAs and two isolated sensors	Second-order difference coarray	One-dimensional method	Weight/leakage indicators
Proposed array	Three closed-form subarrays per branch	Second-order difference coarray	Conditional cross-covariance pairing	Distance-indexed coefficient model

**Table 2:** Analytical geometry metrics of four 23-sensor L-shaped arrays.

Array	One-Branch Normalized Position Set	A/d	$D_{\mathrm{tot}}$	$D_{u}$	[w(1),w(2),w(3)]
L-shaped ULA	{0,1,2,3,4,5,6,7,8,9,10,11}	11	23	23	[11,10,9]
L-shaped coprime array	{0,4,5,8,10,12,15,16,20,25,30,35}	35	59	47	[2,2,2]
LOIAC	{0,4,8,12,16,20,24,28,29,32,34,39}	39	71	65	[1,1,1]
Proposed array	{0,3,6,13,20,27,31,34,35,39,41,43}	43	85	83	[1,2,3]

**Table 3:** Normalized coupling leakage for the four 23-sensor geometries under the common distance-indexed coefficient law.

Array	$|c_{1}|=0.1$	$|c_{1}|=0.3$	$|c_{1}|=0.5$
L-shaped ULA	0.1596	0.4363	0.6286
L-shaped coprime array	0.0779	0.2282	0.3638
LOIAC	0.0618	0.1826	0.2957
Proposed array	0.0639	0.1888	0.3051

## Data Availability

The original contributions presented in this study are included in the article. Further inquiries can be directed to the corresponding author.

## Appendix A. Proof of the Proposition

**Proposition A1.** 
*Let N∈N, N≥4, T=3N, and M=⌈N/2⌉. For the branch position set in (1), the largest central contiguous difference set is $D_{c}=\left\{-L_{c},\ldots ,L_{c}\right\}$, where*

(A1)
$$L_{c}=\left\{\begin{array}{cc} (2N-1)(T-2N+2)-1, & N\geq 4\;\mathrm{is}\;\mathrm{even},\\ (2N-1)(T-2N+2)+N-2, & N\geq 5\;\mathrm{is}\;\mathrm{odd}. \end{array}\right.$$

*Consequently, $D_{u}=2L_{c}+1$, which gives the two parity-dependent expressions stated in Section 2.2.*

**Proof.** The proof has three steps. First, we rewrite the physical set without changing it. Next, we show that two cross-difference families cover every residue modulo 2N−1. Finally, we use the finite sensor-index bounds to determine the attained quotient intervals and the first missing lag. Define a=N−1, b=2N−1, h=Ma, and $g=bN-a^{2}+2Ma$. For the proof only, augment Subarray 2 by the point *h*:

(A2) $${\tilde{S}}_{2}=S_{2}\cup \left\{h\right\}=\left\{h+bj:0\leq j\leq N+1\right\}.$$

Because M≤N−2 for N≥4, h=Ma is already the i=M member of $S_{1}$. Hence this proof-only augmentation adds no physical sensor and changes neither S nor its difference set D=S−S. With $A=S_{1}$, $B={\tilde{S}}_{2}$, and $C=S_{3}$, the three finite sets are

(A3) $$\begin{array}{cc} A & =\{ai:0\leq i\leq N-2\},\\ B & =\{h+bj:0\leq j\leq N+1\},\\ C & =\{g+Nk:0\leq k\leq N-1\}. \end{array}$$

Every integer has a unique representation qb+r, where q∈Z and 0≤r≤b−1. Consider the residues supplied by B−A and C−B:

(A4) $$\begin{array}{cc} R_{BA} & =\{{\left[h-ai\right]}_{b}:0\leq i\leq N-2\},\\ R_{CB} & =\{{\left[g-h+Nk\right]}_{b}:0\leq k\leq N-1\}, \end{array}$$

where ${\left[x\right]}_{b}$ denotes reduction modulo *b*. The residues within each set are distinct because gcd(N,b)=gcd(N−1,b)=1. If one residue occurred in both sets, substitution of $g=bN-a^{2}+2h$ would give i−k≡N−1(mod2N−1). However, −(N−1)≤i−k≤N−2, so neither N−1 nor its congruent value −N is possible. The two disjoint sets contain N−1 and *N* residues, respectively; therefore,

(A5) $$R_{BA}\dot{\cup }R_{CB}=\left\{0,1,\ldots ,b-1\right\}.$$

This residue partition is necessary but not sufficient: coprimality alone does not prove that bounded sensor indices generate a consecutive interval. We therefore retain the finite index limits. For 0≤i≤N−2 and 0≤k≤N−1, define the exact quotient–remainder pairs

(A6) $$\begin{array}{cc} h-ai=b\alpha_{i}+\rho_{i}, & \;\;\;\;\;\;\;\;\;\;\;\;\;\;\;\;0\leq \rho_{i}<b,\\ g-h+Nk=b\beta_{k}+\tau_{k}, & \;\;\;\;\;\;\;\;\;\;\;\;\;\;\;\;0\leq \tau_{k}<b,\\ -\left(g-h+Nk\right)=b\delta_{k}+\zeta_{k}, & \;\;\;\;\;\;\;\;\;\;\;\;\;\;\;\;0\leq \zeta_{k}<b. \end{array}$$

For 0≤t≤2N−3, also write $g+Nt=b\gamma_{t}+\eta_{t}$, $0\leq \eta_{t}<b$, and set $i_{\min}\left(t\right)=\max\left(0,t-N+1\right)$ and $i_{\max}\left(t\right)=\min\left(N-2,t\right)$. For a residue *r*, let $Q_{r}=\left\{q\in Z:qb+r\in D\right\}$. Direct substitution of the bounded indices gives the following finite quotient intervals. For example, $h-ai+bj=b\left(\alpha_{i}+j\right)+\rho_{i}$ with 0≤j≤N+1, so B−A supplies every quotient from $\alpha_{i}$ to $\alpha_{i}+N+1$ at residue $\rho_{i}$. The other three rows follow by the same exact division:

(A7) $$\begin{array}{cccc} B-A:\; & r=\rho_{i}, & q & =\alpha_{i},\ldots ,\alpha_{i}+N+1,\\ C-B:\; & r=\tau_{k}, & q & =\beta_{k}-N-1,\ldots ,\beta_{k},\\ C-A:\; & r=\eta_{t}, & q & =\gamma_{t}-i_{\max}\left(t\right),\ldots ,\gamma_{t}-i_{\min}\left(t\right),\\ B-C:\; & r=\zeta_{k}, & q & =\delta_{k},\ldots ,\delta_{k}+N+1. \end{array}$$

For clarity, each row is an interval of integers and is included in $Q_{r}$ for its displayed residue; no unbounded Bézout representation is used. Substituting M=N/2 for even *N* and M=(N+1)/2 for odd *N*, and then collecting the four finite intervals residue by residue, gives

(A8) $$\begin{array}{ccc} \mathrm{parity} & \mathrm{residue}\;r & \mathrm{guaranteed}\;q\;\mathrm{in}\;Q_{r}\\ N\;\mathrm{even} & 0\leq r\leq b-1 & 0\leq q\leq N+1\\ N\;\mathrm{odd} & 0\leq r\leq N-2 & 0\leq q\leq N+2\\ N\;\mathrm{odd} & N-1\leq r\leq b-1 & 0\leq q\leq N+1 \end{array}$$

The intervals in each residue class meet or overlap at their endpoints; hence they contain no missing quotient within the stated bounds. It follows that all integers in [0,b(N+2)−1] are attained when *N* is even. When *N* is odd, residues 0,…,N−2 are also attained in block q=N+2, extending the interval to b(N+2)+N−2. To establish maximality, rather than only a lower bound, substitute the next candidate into the finite self- and cross-difference families A−A, B−B, C−C, B−A, C−A, and C−B and their negatives. Their bounded index ranges exclude b(N+2) when *N* is even and exclude b(N+2)+N−1 when *N* is odd. Thus, these are the first missing positive lags. Since every difference set is symmetric about zero, the claimed $D_{c}$ and $L_{c}$ follow. Two small cases make the parity result transparent. For N=4, M=2 and direct differencing gives $D_{c}=\left\{-41,\ldots ,41\right\}$, so $D_{u}=83$ and ±42 are absent. For N=5, the ceiling rule gives M=3 and the branch positions are {0,4,8,12,21,30,39,48,53,57,58,63,66,68,73}. Their difference set contains {−66,…,66} but not ±67, so $D_{u}=133$. These examples check the two parity branches; the general result follows from the finite quotient argument above.    □
